# Cognitive functioning and psychosomatic syndromes in a subjective tinnitus sample

**DOI:** 10.3389/fpsyg.2023.1256291

**Published:** 2023-12-20

**Authors:** Daphne Gasparre, Ilaria Pepe, Domenico Laera, Chiara Abbatantuono, Maria Fara De Caro, Alessandro Taurino, Daniele D’Erasmo, Piero Fanizzi, Linda A. Antonucci, Alessandra Pantaleo, Giada Cavallaro, Vito Pontillo, Paolo Taurisano, Nicola Quaranta

**Affiliations:** ^1^Department of Translational Biomedicine and Neuroscience “DiBraiN”, University of Bari “Aldo Moro”, Bari, Italy; ^2^Clinical Psychology Service, Mental Health Department, ASL Taranto, Taranto, Italy; ^3^Department of Education, Psychology, Communication, University of Bari, Palazzo Chiaia-Napolitano, Bari, Italy; ^4^Otolaryngology Unit, Department of Biomedical Sciences, Neuroscience and Sensory Organs, University of Bari Aldo Moro, Bari, Italy

**Keywords:** tinnitus, psychosomatic disorders, cognitive impairment, executive functions, anxiety, depression

## Abstract

**Introduction:**

Tinnitus is the perception of a sound in the absence of any corresponding external sound source. Current research suggests a relationship among emotional, cognitive, and psychosomatic symptoms and the occurrence or maintenance of chronic tinnitus. This study aimed to detect the prevalence and role of psychosomatic conditions, as defined by the Diagnostic Criteria for Psychosomatic Research (DCPR), and cognitive functioning in a group of patients with tinnitus.

**Methods:**

Sixty-two patients with subjective tinnitus and 62 non-tinnitus controls were recruited from the Otorhinolaryngology Unit of the University of Bari. Pure-tone audiometry was performed in all tinnitus subjects, and sound level tolerance was evaluated. Additionally, tinnitus handicap (Tinnitus Handicap Inventory [THI]), psychopathological symptoms (Symptom Checklist-90, Revised [SCL-90-R]), anxiety (State–Trait Anxiety Inventory [STAI-Y1/2]), depression (Beck Depression Inventory [BDI]), cognitive impairment (Mini-Mental State Examination [MMSE]), executive functions (Frontal Assessment Battery [FAB]), and psychosomatic syndromes (DCPR) were evaluated. Parametric and non-parametric tests were used to detect cognitive and symptomatological differences between patients and controls. The predictivity of these factors for tinnitus severity was studied using multiple regression (Backward Elimination). All tests were considered significant at *p* < 0.05 (family wise error corrected for each comparison).

**Results:**

69.4% tinnitus patients met multiple DCPR criteria, compared to 32.3% of controls. Tinnitus patients exhibited elevated rates of illness denial (ꭓ^2^ = 9.02; *p* < 0.009), demoralization (ꭓ^2^ = 8.05; *p* < 0.018), somatization (ꭓ^2^ = 4.92; *p* < 0.063) and functional symptoms (ꭓ^2^ = 5.21; *p* < 0.06) scoring significantly higher on the BDI, STAI-Y1, and STAI-Y2, and SCL-90-R compared to controls. Patients with tinnitus showed lower MMSE scores, compared to controls (*t* = −2.282; *p* < 0.001). No association between tinnitus severity and global cognitive impairment emerged. Conversely, executive function deficits were associated to tinnitus severity. Among the cognitive and psychological factors, only trait anxiety, one or more psychosomatic syndromes, and somatization clusters were strongly correlated with tinnitus severity.

**Discussion:**

Our findings suggest a relationship between tinnitus severity, psychological, psychosomatic symptoms, and frontal impairment. Additionally, the influence of tinnitus on cognitive functions paves the way for integrated, multidisciplinary diagnostic and treatment options for patients. Although preliminary, our findings highlight the importance of early cognitive and psychological screening to improve patients’ quality of life.

## 1 Introduction

Tinnitus is the perception of a sound, usually in the form of a high-pitched tone, ringing, or noise, in the absence of any corresponding external sound source (Baguley et al., 2013) and according to the World Health Organization affects about 278 million people worldwide (Carrera et al., 2022). Objective tinnitus refers to the perception of a sound by both the patient and the examiner and can be caused by turbulent blood flow or myoclonus, subjective tinnitus is a medical symptom characterized by the perception of a phantom ringing, buzzing, and/or hissing sound in the absence of an external sound source only by the patient (De Ridder et al., 2021).

Tinnitus is usually associated with middle and inner ear pathologies, however it can be identified also in subjects with normal hearing (McKee and Stephens, 1992; Monzani et al., 2008). Psychological variables have been reported in the literature as mechanisms contributing to the perpetuation of symptoms (Hazell and Jastreboff, 1990).

Jastreboff and Jastreboff (2018) proposed a model that explains the physiopathology of tinnitus, integrating neurophysiological and psychological features, incorporating, predisposing, precipitating, and maintaining factors (Jastreboff et al., 1996). According to recent updates of this model, tinnitus becomes chronic and decompensated as a consequence of circuit malfunction in a neural network, involving sensory, limbic, and autonomic components (Georgiewa et al., 2006).

Tinnitus, as a subjectively perceived symptom, can cause psychiatric comorbidities such as depression and anxiety. The onset of tinnitus has been shown to lead to anxiety and other negative emotions and tends to alter the way people perceive tinnitus symptoms via the maintenance pathway. Such symptomatology can be detected in 20% of the population with tinnitus (Schutte et al., 2009). Hence, anxiety and depression have been defined as predictors of catastrophic tinnitus (Wallhäusser-Franke et al., 2017).

Most of the current research highlights the role of anxiety and dysfunctional emotion regulation in the maintenance of chronic tinnitus (Trevis et al., 2016). Irritability (28%), somatoform disorders (15%), and behavioral disorders (3%) were the most common comorbidities (Reavis et al., 2020).

Patients who find their tinnitus unacceptable are more likely to try to avoid it (Hesser et al., 2012), and this avoidance strategy may create a vicious cycle, increasing their perception of the disturbing stimulus (Hayes et al., 2006).

Hiller et al. (1997), conducted an international study initiated by WHO (*n* = 1,275). Their findings indicated a higher prevalence of tinnitus (42%) in patients diagnosed with somatization disorder. The authors posited that a potential association between tinnitus and somatization might arise from shared mechanisms involving autonomic arousal; the complex relationship between tinnitus and psychosomatic diseases (such as somatization) does not allow us to make inferences regarding direct causality. A set of psychosomatic reactions preceding tinnitus onset could play a significant role in the course and management of the disease. Conversely, tinnitus itself could lead to the onset of psychosomatic illness or exacerbation of pre-existing psychosomatic conditions.

Following a holistic approach, psychosomatic medicine has developed several clinometric tools for assessing psychosocial variables in the medical disease setting. Porcelli and Guidi (2015) introduced, with an international team of researchers, the Diagnostic Criteria for Psychosomatic Research (DCPR), a structured interview that contains a set of criteria for identifying 12 psychosomatic syndromes and provides an operational strategy for assessing psychosocial variables with prognostic and therapeutic implications in clinical settings. Indeed, this tool accounts for abnormal illness behavior, various modalities of somatization, irritability, demoralization, and alexithymia.

Further, the impact of chronic tinnitus on patients’ lives also affects cognitive function, and many cross-sectional or longitudinal cohort studies have documented that hearing impairment is associated with cognitive decline (Park, 2016). Changes in the central auditory pathway, together with neuroplastic reorganization within the auditory cortex, thalamus, and structures of the limbic and paralimbic circuits (Xu et al., 2019), have led some authors to hypothesize a possible relationship between tinnitus and cognitive impairment, with a positive correlation with tinnitus severity (Wang et al., 2018; Brueggemann et al., 2021).

Recent studies have also shown that tinnitus intensity and perception are correlated with lower performance on tasks involving the deployment of cognitive functions (Neff et al., 2021). Just as cognitive and perceptual loads are likely to have a significant influence on both tinnitus perception and emotional well-being, cognitive resource overload in chronic tinnitus leads to failures in executive control tasks in accordance with the so-called “load theory” (Khan and Husain, 2020).

As a result, patients with severe tinnitus may experience higher cognitive deficits than controls, with an obvious decrease in quality of life and work productivity (Wang et al., 2018). Additionally, tinnitus becomes more prevalent as individuals grow older (Jafari et al., 2019), but there is no direct association still needs to be further investigated. To this end, the MMSE was used to estimate global cognitive functioning, whereas the FAB was used as a screening test to assess executive function among patients with tinnitus. Executive attention refers to the ability to regulate our responses, engaging and disengaging a stimulus and switching to a different stimulus. Recent studies showed that tinnitus is associated with poorer executive function, processing speed, general short-term memory, and general learning and retrieval. Narrow cognitive domains of inhibition and shifting and learning and retrieval were also associated with tinnitus (Tegg-Quinn et al., 2016).

Accordingly, the first aim of this study was to explore the prevalence of psychosomatic conditions, as defined by the DCPR in the tinnitus sample matched with a control sample. In particular, we investigated the potential association between the psychosomatic symptoms and the presence of subjective tinnitus, comparing patterns between the tinnitus sample and healthy participants.

In this regard, we were also interested in examining the relationship between psychosomatic states and tinnitus severity; specifically, whether tinnitus severity could be predicted based on the presence of somatic-psychological symptoms, as outlined by DCPR. The second aim was to investigate the relationship between tinnitus condition and cognitive performances in both tinnitus patients and controls. We assessed the global cognitive functioning by means of the MMSE (Measso et al., 1993; Magni et al., 1996) and the executive functions using the FAB (Dubois et al., 2000) in relation to tinnitus severity. In this perspective, we also wanted to consider the impact that the severity perception of tinnitus, evaluated by Tinnitus Handicap Inventory (THI), might have on cognitive performances.

## 2 Materials and methods

Sixty-two patients (51.6% female; mean age 53.7 years) with chronic tinnitus, defined as the presence of tinnitus for at least 6 months (mean duration: 6.1 ± 8 years; range: 0.3–35 years) were recruited from the Otorhinolaryngology Unit of the University of Bari. Sixty-two non-tinnitus control participants, matched by age and sex (51.6% female; mean age 53.6 years), were also recruited.

The sample size was projected using G Power 3.1.9.7. (Faul et al., 2007), which took into account expected effect sizes, desired levels of statistical significance, and potential attrition rates. Accordingly, we aimed for a sample size of 64 participants. Before carrying out a comprehensive assessment of participants, 65 volunteers were recruited; three individuals were subsequently excluded because of their reduced sound tolerance, primarily attributable to pure misophonia without concurrent tinnitus. These individuals were consequently referred for sound desensitizing therapy. No additional eligible patients who expressed a willingness to participate in the study were identified. Hence, the final sample size in our study was 62 participants, which is slightly below our initial target.

Institutional Review Board approval was obtained from the University of Bari General Hospital, and the study was conducted in accordance with the Declaration of Helsinki. All clinical participants were informed about the goals of the study and the collection of data, and they were reassured of the confidentiality and anonymity of the information provided. All the participants provided informed consent. Chronic tinnitus was diagnosed by two well-trained otolaryngologists based on criteria published by Shulman and Farhadi (Shulman et al., 2009; Farhadi et al., 2010). Exclusion criteria were either acute or chronic pathology of the external auditory canal or middle ear, Eustachian Tube Dysfunction, neurological disorders (e.g., dementia), and psychological and psychiatric diseases. Dementia was diagnosed according to the *Diagnostic and Statistical Manual of Mental Disorders* (DSM-5) criteria (American Psychiatric Association, 2013) after a clinical examination that included cognitive testing and an interview with a caregiver. All tinnitus subjects underwent brain and inner ear magnetic resonance imaging (MRI).

### 2.1 Assessment instruments

#### 2.1.1 Clinical assessment

Tinnitus subjects underwent pure-tone conventional audiometry (0.125–8.0 kHz) in a standard sound-proof room (Madsen Astera with TDH 39 Headphones, Natus Medical Denmark) and impedance audiometry (Impedance Meter Resonance). Hearing loss (HL) was defined as an average hearing threshold for frequencies between 0.5 and 2 kHz (PTA = Pure Tone Average) greater than 30 dB HL.

The Decreased Sound Tolerance (Loudness Discomfort Level) was determined for tones of 0.5, 1, 2, and 4 kHz, using the same equipment. It was defined as absent if the sound was perceived as uncomfortably loud above 95 dB, mild if it was between 80 and 90 dB at two or more frequencies, moderate if it was between 65 and 75, and severe if it was less than 60. The test was performed twice to distinguish between misophonia and hyperacusis. In the context of sample recruitment, it is pertinent to differentiate between hyperacusis and misophonia, which are recognized as two expressions of reduced sound tolerance. Hyperacusis is a well-recognized and diagnosable pathological condition characterized by a heightened sensitivity to sounds caused by a hearing loss from a cochlear receptor lesion, whereas misophonia, not yet formally recognized as a pathological condition, primarily denotes intolerance to specific sounds, typically emitted by third parties, be they people or objects. Misophonia can sometimes lead to negative emotional and physical reactions (Henry et al., 2022) that eventually manifest phonophobia (Hazell and Jastreboff, 1990). Consequently, individuals diagnosed with hyperacusis are part of the study sample based on their diagnosed hearing disorder. On the contrary, individuals with misophonia, which are not normoacusical acufenopathic subjects but only healthy subjects with reduced sound tolerance, were excluded from our study and referred for sound desensitizing therapy.

The intensity of sound was increased gradually at each frequency until discomfort was reported, at which time the measurement was stopped, and the sound level in dB was recorded (Snow, 2004).

The patients were then interviewed regarding tinnitus characteristics, duration, and laterality. The severity of tinnitus was assessed using the Italian version of the Tinnitus Handicap Inventory (THI) (Newman et al., 1996; Monzani et al., 2008). The THI (Cronbach’s α = 0.92) is a 25-item questionnaire with three possible answers: 4-point (“yes”), 0-point (“no”), or 2-point (“sometimes”). The total scores yielded the classification of tinnitus annoyance into five grades: slight (0–16), mild (18–36), moderate (38–56), severe (58–76), or catastrophic (78–100) (McCombe et al., 2001).

#### 2.1.2 Psychological assessment

Anxiety was assessed using the State–Trait Anxiety Inventory (STAI-Y) (Pedrabissi and Santinello, 1989), which is divided into two sections: (1) STAI-Y1, which assesses current feelings of apprehension, tension, nervousness, and worry (state anxiety); and (2) STAI-Y2, which assesses persistent anxiety traits. Each section consisted of 20 items, with a total score ranging from 20 to 80. The patient is asked to answer marking the most representative option on a 4-point Likert scale. Once the inverted items have been corrected, the scores obtained are added together and the overall figure for each scale is related to 4 different anxiety thresholds (0–52 = Normal; 53–62 = Mild; 63–70 = Moderate; ≥71 = Severe). Widely used in clinical contexts, the STAIs are, moreover, considered the gold standard for the assessment of anxiety, since they produce valid and reliable results (Cronbach’s α is 0.91–95 for STAI-Y1 and 0.85–90 for STAI-Y2).

Depressive symptoms were assessed using the Beck Depression Inventory-II (BDI-II) (Beck et al., 1996; Sica and Ghisi, 2007). The questionnaire (Cronbach’s α = 0.89). presents 21 items. The patient, referring to 4 weeks prior to administration, could choose among 5 possible answers. Each item represents a “symptom attitude type, based on a four-point scale. The total score ranged from 0 (minimal depression) to 63 (severe depression). The sum of the scores obtained is compared with 4 levels of impairment (0–9 = Normal; 10–14 = Mild; 15–23 = Moderate; ≥24 = Severe).

Psychopathological symptoms were evaluated using the Italian version of the Symptom Checklist-90-R (SCL-90-R); (Derogatis, 1983; Prunas et al., 2012). The SCL-90-R (Cronbach’s α = 0.93) is a widely used 90-item self-administered questionnaire intended to measure the self-reported severity of a patient’s symptoms over the previous 7 days. Each item is rated on a 5-point Likert scale ranging from “Not at all” (0) to “Extremely” (4). The checklist consisted of nine subscales and three global distress indices. Nine subscales are divided into several categories: (1) Somatization (SOM), (2) Obsessive-compulsive (O–C), (3) Interpersonal sensitivity (I-S), (4) Depression (DEP), (5) Anxiety (ANX), (6) Hostility (HOS), (7) Phobic anxiety (PHOB), (8) Paranoid ideation (PAR), and (9) Psychoticism (PSY). The general indices were Global Severity (GSI), Positive Symptom Total (PST), and Positive Symptom Distress (PTSD) indices. In our study, we focused on the GSI score as the single best indicator of the current level or depth of a patient’s disorder. GSI combines information concerning the number of symptoms reported with the intensity of perceived distress.

#### 2.1.3 Psychosomatic assessment

Psychosomatic syndromes were investigated using the Structured Interview for DCPR (Rafanelli et al., 2003; Porcelli and Sonino, 2007). The structured face-to-face interview was composed of 58 items scored in a yes/no format and evaluated the presence of one or more of 12 psychosomatic syndromes (alexithymia, type A behavior, irritable mood, demoralization, disease phobia, thanatophobia, health anxiety, illness denial, Functional Somatic Symptoms [FSS] secondary to a psychiatric disorder, persistent somatization, conversion symptoms, and anniversary reaction). The DCPR interview has excellent inter-rater reliability and constructs and predictive validity for treatment outcomes and psychosocial functioning (Galeazzi et al., 2004). The interview has demonstrated considerable interrater concordance for all 12 syndromes, with all κ values exceeding 0.61, and nearly impeccable agreement for 9 of these syndromes, with κ values surpassing 0.81. The interviewer was a clinical psychologist trained in the administration of the Structured Interview for DCPR.

#### 2.1.4 Cognitive assessment

To assess executive functioning, we used the FAB (Dubois et al., 2000; Appollonio et al., 2005). The FAB (Cronbach’s α = 0.78) is divided into six subtests: (1) Similarities in which the domain of abstract reasoning/conceptualization by presenting pairs of objects from the same semantic category is examined, (2) Phonological Verbal Fluency in which self-organized strategy and shifting (i.e., mental flexibility), generating as many words as possible beginning with a given letter is assessed, (3) Motor Series in which the domain of motor programming and planning by carrying out Luria’s “fist-edge-palm” series is examined, (4) Conflicting Instructions in which the domain of sensitivity to interference is examined. Subjects must provide an opposite response to the examiner’s alternating signal; (5) Go-No Go Task, in which the ability to withhold a response, inappropriately induced by both previous learning and concomitant sensory information, is examined, and the domain of inhibitory control is also investigated; and (6) Prehension Behavior, in which the ability to spontaneously inhibit prehension is assessed.

The score for each subtest may vary from 0 to 3, with a score of zero given when the subject failed to provide an answer or responded inappropriately. Thus, a maximum score of 18 was obtained, and administration of the entire battery required approximately 10 min.

Cognitive functioning was assessed using the MMSE (Measso et al., 1993; Magni et al., 1996), a screening instrument (Cronbach’s α = 0.89) widely used to assess cognitive functioning in adults, including temporal and spatial orientation, verbal and drawing functions, memory, calculation, concentration, and attention (Folstein et al., 1975).

### 2.2 Analyses

The statistical package SPSS (Chicago, IL) 24.0 for Windows was used for all analyses.

Data were checked for normality using the Shapiro–Wilk test and homogeneity of variance with Levene’ s test. We used Student’s *t*-test for independent samples and the χ^2^ test to compare demographic variables, clinical characteristics, cognitive functioning, and psychosomatic syndromes between the tinnitus and control groups. Global cognitive functioning, as well as executive functioning, was also assessed for tinnitus severity. Cohen’s d was used to assess effect size. Student’s *t*-test was used to compare anxiety, depression, and psychopathological symptoms between the tinnitus and control groups, and between tinnitus patients with and without the 12 psychosomatic syndromes. Where the normality assumption was not met, Mann–Whitney U-test for independent samples was used instead. To investigate the role of individual variables in explaining tinnitus-related impairment (THI) in the tinnitus group, backward multiple regression analyses were performed, with THI as the dependent variable and cognitive, psychological, and clinical variables as predictors.

All analyses performed are exploratory. For all statistical analyses, *p*-values were considered significant at *p* < 0.05 (adjusted *p* value of 0.05 after Benjamini–Hochberg correction). According to best research practices, we confirm that the data used in this study is part of a larger investigation. The variables as well as statistical analyses used in this study were chosen after data collection but before looking at the data.

## 3 Results

### 3.1 Clinical and socio-demographic variables

Sixty-two subjects with tinnitus, including 26 affected by bilateral tinnitus and 36 with unilateral tinnitus, were evaluated. Among the participants with tinnitus, nine (14.5%) had a primary school diploma, 20 (32.3%) had a first-level secondary school diploma, 18 (29%) had a second-level secondary school diploma, and 15 (24.2%) had a bachelor’s degree. Among the controls nine (14.5%) had a primary school diploma, 25 (40.3%) had a first-level secondary school diploma, 20 (32.3%) had a second-level secondary school diploma, and eight (12.9%) had a bachelor’s degree.

Brain MRI were normal in all subjects with tinnitus. Tinnitus was perceived as a tone in 31 and as a noise in the remaining 31 cases. Decreased sound-level tolerance was reported in 16 cases (26%) and was distributed between mild and moderate levels. Among the 62 selected patients, 12 were normoacusical patients, who presented a mean pure tone better than 30 db (17.7%), two of which have reduced sound tolerance from associated misophonia; sensorineural HL was present in the remaining 50 (80.7%) cases, 14 of which have reduced sound tolerance (recruitment hyperacusia).

HL was bilateral in 36 patients (61.7%) and unilateral in 14 patients (22.6%). HL was flat in 26 patients (41.9%), with low frequencies in 11 (17.7%) and high frequencies in 13 (21%). The average THI was 53.08 (SD = 25.59). The THI grade was slight in five cases (8.1%), mild in 16 cases (25.8%), moderate in 14 cases (22.6%), severe in 12 cases (19.3%), and catastrophic in 15 cases (24.2%).

There were no differences in age, sex, educational level, or occupation between the clinical and control groups (*t* = 1.036; *p* = 0.302). No statistically significant differences were found between female and male participants in any of the clinical, psychological, or psychosomatic variables, except for a higher prevalence of at least one DCPR diagnosis in the female subgroup (χ^2^ = 3.78; *p* < 0.001).

### 3.2 Psychological variables

The psychological and cognitive variables of the two groups are presented in Table 1. Patients with tinnitus showed higher scores on the BDI, STAI-Y1, and STAI-Y2, as well as higher mean scores on all the SCL-90-R subscales, except for Interpersonal Sensitivity (although the mean score was barely above the conventional threshold for statistical significance).

**Table 1:** Psychological and cognitive variables both in the tinnitus and control groups.

	Tinnitus group (*n* = 62)	Control group (*n* = 62)	*t*	df	Adjusted *p*-value	Cohen’s *d*
STAI-Y1	39.60 ± 11.08	33.40 ± 8.56	3.454	120	**0.001**	0.625
STAI-Y2	42.40 ± 10.48	34.94 ± 8.12	4.375	120	**0.001**	0.792
Beck depression inventory	11.6 ± 8.12	5.47 ± 5.13	4.996	120	**0.001**	0.909
**SCL-90-R**						
Somatization	57.80 ± 16.65	46.68 ± 7.34	4.808	121	**0.001**	0.867
Obsessive-compulsive	56.90 ± 13.62	46.24 ± 7.34	5.347	121	**0.001**	0.964
Interpersonal sensitivity	52.90 ± 12.77	48.98 ± 8.83	1.968	121	0.055	0.355
Depression	60.80 ± 15.61	45.94 ± 9.93	6.295	121	**0.001**	1.135
Anxiety	58.90 ± 14.26	46.90 ± 10.43	5.333	121	**0.001**	0.962
Hostility	51.20 ± 10.63	45.31 ± 9.54	3.226	121	**0.003**	0.582
Phobic anxiety	57.80 ± 15.62	49.50 ± 8.07	3.721	121	**0.001**	0.671
Paranoid ideation	52.90 ± 11.92	47.26 ± 9.00	2.971	121	**0.005**	0.536
Psychoticism	59.30 ± 15.36	49.53 ± 9.18	4.295	121	**0.001**	0.775
Global severity index	58.3 ± 15.08	46.56 ± 8.06	5.377	121	**0.001**	0.970
MMSE	27.40 ± 2.35	28.65 ± 1.50	1299*	122	**0.001**	0.658
FAB	15.80 ± 2.04	15.81 ± 1.96	−0.123	120	0.902	−0.022

t = t-statistic; t* = Mann–Whitney U df = degrees of freedom; Adjusted *p*-value = *p*-value adjusted by Benjamini- Hochberg.

STAI-Y2, State–Trait Anxiety Inventory - Y2; STAI-Y1, State–Trait Anxiety Inventory – Y1; SCL-90-R, Symptom Checklist-90-Revised; MMSE, Mini Mental State Examination; FAB, Frontal Assessment Battery. The values statistically significant are provided bold in the table.

### 3.3 Psychosomatic assessment

The percentages and frequencies of DPCR in both tinnitus and control groups are shown in Table 2. In the tinnitus sample the 69.4% met multiple criteria for DCPR conditions (vs. 32.3% controls), and 30.6% showed zero to one symptom in DCPR (vs. 67.7% controls). Hence 43 tinnitus patients showed more than one psychosomatic symptoms.

**Table 2:** Percentages and frequencies of the Diagnostic Criteria for Psychosomatic Research (DCPR).

		Tinnitus group (*n* = 62)	Control group (*n* = 62)	ꭓ^2^	Adjusted *p*-value
Abnormal illness behavior	Health anxiety	12.9% (8)	6.5% (4)	1.48	0.378
	Disease phobia	4.8% (3)	1.6% (1)	1.03	0.393
	Thanatophobia	8.1% (5)	6.5% (4)	0.12	0.785
	Illness denial	29.0% (18)	8.1% (5)	9.02	**0.009**
Somatization syndromes	Functional symptoms	8.1% (5)	0.0% (0)	5.21	0.062
	Persistent somatization	11.5% (7)	1.6% (1)	4.92	0.063
	Conversion symptom	4.8% (3)	4.8% (3)	0.00	1.00
	Anniversary reaction	8.1% (5)	6.5% (4)	0.12	0.785
Irritability	Type A behavior	17.7% (11)	8.1% (5)	2.58	0.216
	Irritable mood	16.1% (10)	9.7% (6)	1.15	0.393
Demoralization	Demoralization	19.4% (12)	3.2% (2)	8.05	**0.018**
Alexithymia	Alexithymia	8.1% (5)	3.2% (2)	1.36	0.378
DCPR_0	Up to one DCPR	30.6% (19)	67.7% (42)	17.07	**0.003**
DCPR_1	Two to several DCPR	69.4% (43)	32.3% (20)	17.07	**0.003**

ꭓ^2^ = chi-squared (chi-square) statistic; Adjusted *p*-value = *p*-value adjusted by Benjamini- Hochberg.

STAI-Y2, State–Trait Anxiety Inventory - Y2; DCPR, Diagnostic Criteria for Psychosomatic Research; DPCR_0, zero to one Diagnostic Criteria for Psychosomatic Research; DPCR_1, from two to several Diagnostic Criteria for Psychosomatic Research. The values statistically significant are provided bold in the table.

The five most prevalent DCPR syndromes were illness denial (29%), demoralization (19.4%), type A behavior (17.7%), irritable mood (16.1%), and health anxiety (12.9%).

When considering patients with tinnitus, we found a significantly higher prevalence in two out of 12 psychosomatic syndromes: (1) illness denial (*p* = 0.009), (2) demoralization (*p* = 0.018). No statistically significant correlations emerged concerning tinnitus severity as measured by the THI and three specific dimensions such as Irritability, Somatization, and Abnormal Illness Behavior (AIB).

Among tinnitus participants, we compared psychopathological symptoms and sociodemographic variables in patients with and without psychosomatic syndrome. Patients with persistent somatization showed higher levels of trait anxiety (*t* = −2.994, *p* = 0.004), state anxiety (*t* = −2.028, *p* = 0.047), depression (*t* = 3.719, *p* < 0.001), and psychopathological symptom severity (*t* = 3.417, *p* < 0.001). Patients with type A behavior were younger (*t* = 3.528, *p* < 0.001) than controls. Irritable-mood patients were younger (*t* = 2.607, *p* = 0.012) and scored higher on the GSI (*t* = −2.452, *p* = 0.017). Patients with demoralization showed higher levels of state anxiety (*t* = −2.731, *p* = 0.008), trait anxiety (*t* = −2.289, *p* = 0.026), and psychopathological symptoms (*t* = −2.517, *p* = 0.015). Participants with at least one DCPR diagnosis compared with those without DCPR diagnosis reported a greater impact of tinnitus in daily life (*t* = −2.829, *p* = 0.006), higher severity of psychopathological symptoms (*U* = 191, *p* = 0.006), and higher scores on depression (*t* = −2.721, *p* = 0.006). No differences were found between patients with and without DCPR syndrome in terms of sex and hearing loss.

### 3.4 Cognitive assessment

As shown in Table 1, no statistically significant differences were found in the FAB scores between participants with tinnitus and control participants (*t* = 0.354; *p* > 0.05). Nevertheless, when studying the association between tinnitus severity and frontal deterioration among patients with tinnitus, a statistically significant association emerged between the deficit category at FAB and the degree of severe and catastrophic severity of THI (*p* = 0.026) (Table 3).

**Table 3:** Percentages and frequencies of cognitive variables with respect to the degree of severity of subjective tinnitus (THI).

	Degree of severity of subjective tinnitus (THI)					
Cognitive variables	Slight	Mild	Moderate	Severe	Catastrophic	Total
***FAB***	*Fisher exact test: p-value = 0.026 < α = 0.05*					
Deficit	40.0 (2)	100.0 (16)	80.0 (12)	90.9 (10)	66.7 (10)	80.6 (50)
Borderline	0.0 (0)	0.0 (0)	6.7 (1)	0.0 (0)	6.7 (1)	3.2 (2)
Within the normal range	60.0 (3)	0.0 (0)	13.3 (2)	9.1 (1)	26.7 (4)	16.1 (10)
Total	100.0 (5)	100.0 (16)	100.0 (15)	100.0 (11)	100.0 (15)	100.0 (62)
***MMSE***	*Fisher exact test: p-value = 0.876 > α = 0.05*					
Deficit	80.0 (4)	75.0 (12)	66.7 (10)	63.6 (7)	53.3 (8)	66.1 (41)
Borderline	20.0 (1)	25.0 (4)	33.3 (5)	36.4 (4)	40.0 (6)	32.3 (20)
Within the normal range	0.0 (0)	0.0 (0)	0.0 (0)	0.0 (0)	6.7 (1)	1.6 (1)
Total	100.0 (5)	100.0 (16)	100.0 (15)	100.0 (11)	100.0 (15)	100.0 (62)

MMSE, Mini Mental State Examination; FAB, Frontal Assessment Battery.

While significant differences were found in MMSE scores between the tinnitus group and the control group (*t* = −2.282; *p* < 0.001; Table 1), no association was found between equivalent MMSE scores and tinnitus-related severity level (*p* = 0.876; Table 3).

### 3.5 Regression analysis

To assess the independent contribution of each factor in explaining the extent of tinnitus-related impairment (THI), backward multiple regression analysis was performed with THI as the dependent variable.

Cognitive variables (FAB; MMSE), psychological variables (STAI-Y1, STAI-Y2, BDI, GSI, DCPR syndromes, DCPR Abnormal Illness Behavior cluster, DCPR Somatization cluster, DCPR Irritability cluster, DCPR_0 = absence, DPCR_1 = at least one disorder), and clinical variables (hearing loss, tinnitus duration, and laterality) were predictors. However, by including all other predictors in your model, neither SCL-90-scores, BDI-scores, FAB nor MMSE-values predicted THI-levels.

The scatter plot of the un-normalized predictive value (PRE_1) and studentized residual value (SRE_1) showed a linear relationship between the dependent variable (THI) and all independent variables in the multiple linear regression model. The predicted probability plot (PP) determined that the residuals were normally distributed and homoscedastic. Regression tolerance was >0.1 and multicollinearity was not detected.

As shown in Table 4, an *R*-value of 0.653 indicated a good level of THI prediction. Our model yielded an adjusted *R*^2^ value of 0.395, indicating that nearly 40% of the variance in tinnitus-related impairment is accounted for by the selected independent variables. Cohen’s (1988) guidelines classify this as a substantial effect size, underscoring the significance of our findings and highlighting the robust explanatory power of our model. The regression model was significant with *F* = 11.424 (*p* < 0.001; adjusted *R*^2^ = 0.39). Among the independent variables included in the model, four had significant effects on the THI (*p* < 0.05). Specifically, higher trait anxiety (*B* = 0.53; *t* = 5.06; *p* < 0.005) and the presence of one or more psychosomatic syndromes (*B* = 0.35; *t* = 3.20; *p* < 0.005), were statistically associated with higher scores in the THI, as well as the absence of persistent somatization, rather than the presence were statistically predictive of lower scores in the THI (*B* = −0.38; *t* = −3.20; *p* < 0.005). The results are presented in Table 5.

**Table 4:** Backward multiple regression analyses predicting tinnitus-related impairment (THI) from sociodemographic, cognitive, psychological, and clinical variables of Initial model.

Predicting THI	*B*	SD	β	*t*	*p*
(constant)	54.012	42.888		1.259	0.559
Tinnitus duration	0.015	0.029	0.059	0.520	0.727
MMSE_	−1.438	1.315	−0.136	−1.094	0.559
FAB	−0.925	1.445	−0.077	−0.640	0.700
AIB	−2.195	7.716	−0.044	−0.284	0.777
DCPR_SOMATIZATION CLUSTER	−19.652	6.851	−0.345	−2.868	0.074
IRRITABILITY	−4.474	6.987	−0.079	−0.640	0.700
STAI-Y1	0.121	0.299	0.054	0.404	0.751
STAI-Y2	0.820	0.385	0.342	2.245	0.118
BDI	0.468	0.509	0.154	0.921	0.621
DCPR_0	37.042	14.478	0.651	2.559	0.083
DCPR_1	−17.963	15.228	−0.334	−1.180	0.559
	Model summary				
Initial model	*R*	*R* ^2^	Adjusted *R*^2^		
	0.698	0.487	0.380		

B = Unstandardized coefficient; SD = Standard Deviation, β = Standardized coefficient; t = t-statistic; p = *p*-value; R = correlation coefficient; R-square = coefficient of determination, Adjusted R-Square = coefficient of determination adjusted for the number of independent variables.

THI, Tinnitus Handicap Inventory; MMSE, Mini-Mental State Examination; FAB, Frontal Assessment Battery; AIB, Abnormal Illness Behavior; DCPR, Diagnostic Criteria for Psychosomatic Research; STAI-Y1, State–Trait Anxiety Inventory – Y1; STAI-Y2, State–Trait Anxiety Inventory - Y2; BDI, Beck Depression Inventory; DPCR_0, absence of persistent somatization rather than the presence.

**Table 5:** Backward multiple regression analyses predicting tinnitus-related impairment (THI) from sociodemographic, cognitive, psychological, and clinical variables of Final model.

Predicting THI	*B*	SD	β	*t*	Adjusted *p*-value
(Constant)	54.012	0.252		1.259	0.405
DCPR_SOMATIZATION CLUSTER	1.276	0.252	0.533	5.06	0.002
STAI-Y2	−21.87	6.29	−0.384	5.06	<0.001
DCPR_0	19.83	6.20	0.348	3.20	0.003
	Model summary				
Final model	*R*	*R* ^2^	Adjusted *R*^2^		
	0.653	0.426	0.395		

B = Unstandardized coefficient; SD = Standard Deviation, β = Standardized coefficient; t = t-statistic; p = *p*-value; R = correlation coefficient; R-square = coefficient of determination, Adjusted R-Square = .coefficient of determination adjusted for the number of independent variables.

THI, Tinnitus Handicap Inventory; STAI-Y2, State–Trait Anxiety Inventory - Y2; DCPR, Diagnostic Criteria for Psychosomatic Research; DPCR_0, absence of persistent somatization rather than the presence.

## 4 Discussion

In this study, our aims was to investigate a cohort of patients afflicted by subjective tinnitus. Our primary objective was to examine the presence of psychosomatic patterns and potential cognitive deficits within this patient population.

Chronic tinnitus condition is often associated with somatoform disorders; even patients with somatoform disorders sometimes complain of tinnitus; a vicious cycle may be created between the two conditions. Tinnitus may be a form of somatoform disorder. Tinnitus may be otogenic in origin or psychogenic (somatoform) in origin. There is little information in terms of the etiopathogenesis of these disorders. Somatization, depression, obsessions, and irritability often accompany tinnitus (Salviati et al., 2014).

Drawing upon the extensive body of literature documenting the probable impact of tinnitus on both the psychological well-being of patients and their cognitive capabilities, our research ventured beyond mere symptom assessment. Instead, we undertook a comprehensive analysis encompassing the nuanced evaluation of symptom intensity. This approach recognized the individualized perception of this auditory ailment and its potential far-reaching consequences on cognitive and psychological functions. Consequently, our investigation sought to elucidate the intricate interplay between somatic and cognitive dimensions, acknowledging the profound implications of these interactions on the overall quality of life experienced by individuals grappling with tinnitus.

The Diagnostic Criteria for Psychosomatic Research (DCPR) overcome some of the shortcomings of the psychiatric nosography system and represent a diagnostic framework aimed at translating psychosocial variables derived from psychosomatic research into operational tools whereby individual patients can be identified (Porcelli and Rafanelli, 2010). DCPR contains a set of criteria for identifying 12 psychosomatic syndromes, and a large body of literature has documented its prognostic role in the field of physical diseases (Porcelli and Guidi, 2015).

More than half of the patients with tinnitus met the criteria for at least one DCPR diagnosis, and we found a significantly higher prevalence in four out of the12 psychosomatic syndromes: (1) illness denial, (2) demoralization, (3) persistent somatization, and (4) functional symptoms. Patients with one or more DCPR diagnoses reported a greater impact of tinnitus on daily life and showed a worse psychopathological profile.

DCPR syndrome of disease denial was diagnosed three times more frequently in the tinnitus group than in the control group. Denial of the burden of physical illness may be an adaptive coping mechanism under certain circumstances and levels. Patients who find their tinnitus unacceptable are more likely to try to avoid it (Hesser et al., 2012), and this avoidance strategy may create a vicious cycle, increasing their perception of the disturbing stimulus they are trying to avoid (Hayes et al., 2006).

In our sample, patients with tinnitus had six times higher DCPR demoralization than controls. Demoralization, a state of mind characterized by an inability to cope with problems, hopelessness, and helplessness (Frank, 1974), can dramatically increase the subjective perception of being overburdened by stressful demands. Demoralization and major depression can be considered distinct phenomena on clinical grounds. Unlike patients with depression, patients with dementia often do not exhibit a full set of neurovegetative symptoms, and mood reactivity is usually preserved.

In addition, demoralized patients may experience hope and pleasure when adversity is overcome (De Figueiredo, 2013). Demoralization by DCPR has been shown to be prevalent in healthy participants, ranging from 2 to 5%, and a prevalence of 30% in medical settings (Tecuta et al., 2015). Patients with tinnitus might adopt an existential stance that distorts them from the challenges of the disease, leading to maladaptive coping strategies and negative emotions that might reinforce the perception of tinnitus (Trevis et al., 2016).

In this study, the tinnitus cluster was characterized by the presence of functional symptoms and persistent somatization, and the DCPR Somatization cluster predicted tinnitus severity. Somatization has been defined (Lipowski, 1987) as the tendency to experience psychological distress through physical symptoms and to seek help for them. The prevalence of the DCPR category of persistent somatization along with functional symptoms is low in individuals in the community (2–3%) but is high in various medical settings (Porcelli and Rafanelli, 2010). Our results are in line with previous research in which chronic tinnitus has been associated with greater tendencies for somatization and perceived pain (Trevis et al., 2016; Wallhäusser-Franke et al., 2017). Several studies in literature have shown that the constant awareness of tinnitus often causes considerable distress. Tinnitus, as a subjectively perceived symptom, can cause comorbidities such as depression and anxiety. Such symptomatology can be detected in 20% of the population with tinnitus (Schutte et al., 2009). In addition, chronic tinnitus is very common in the adult population and its impact on psychological well-being could be so severe that it has been identified as a risk factor for suicide in the elderly (Johnston and Walker, 1996).

It is imperative to acknowledge the potential convergence or overlapping characteristics among subdomains within the SCL-90-R, including aspects such as anxiety, depression, and somatization. This overlap should be duly considered, especially concerning its relevance and implications when compared with more specialized assessment tools like the STAI-Y, BDI, and DCPR.

In this study, we delved into associations using as predictors of tinnitus severity different measures assessed in patients focusing especially on multidimensionality of DCPR to better grasp somatization aspects that could have been of interest based on the study aims. Even if all these aspects should be considered for patient assessment, we proceeded stepwise answering our research questions proceeding from a general exploration to a more specific one (e.g., focusing on different DCPR subscales that could have offered a more precise identification of psychosomatic components lacking in recent tinnitus research). Hence, our results highlight the involvement of a diverse array of symptoms, rather than a specific symptom, in the syndromic picture of tinnitus, consistent with the mentioned literature.

Our results support the hypothesis that patients with tinnitus show a worse psychopathological pattern than non-clinical subjects. The multiple regression model showed that THI was predicted by trait anxiety. According to the most recent meta-analysis of psychological functioning in chronic tinnitus, depressive and anxiety symptoms are more frequent in patients with severe tinnitus (Neff et al., 2021).

In this study, we delved into associations using as predictors of tinnitus severity different measures assessed in patients focusing especially on multidimensionality of DCPR to better grasp somatization aspects that could have been of interest based on the study aims. Even if all these aspects should be considered for patient assessment, we proceeded stepwise answering our research questions proceeding from a general exploration to a more specific one (e.g., focusing on different DCPR subscales that could have offered a more precise identification of psychosomatic components lacking in recent tinnitus research). Hence, our results highlight the involvement of a diverse array of symptoms, rather than a specific symptom, in the syndromic picture of tinnitus, consistent with the mentioned literature. Indeed in this study, individuals with pre-existing psychiatric diagnoses were excluded, to maintain a more targeted investigation of the psychological symptoms that could be involved in tinnitus as a psychological discomfort and a risk issue, without a disorder diagnosed on DSM criteria.

Severe tinnitus may cause psychological distress as a reaction to overwhelming symptoms. In contrast, the presence of depression and anxiety may reduce tinnitus tolerance, resulting in a somatosensory loop with increased selective attention to phantom sounds. The involvement of emotional factors in chronic tinnitus has been confirmed by imaging studies (Husain, 2016), which have highlighted the role of the limbic system (mainly the amygdala, parahippocampus, and insula) in the pathophysiology of tinnitus.

The use of psychological inventories and structured interviews like the DCPR allowed us to capture aspects of psychological distress, given the psychological burden of tinnitus in daily life. Previous studies remarked on the importance of integrating cognitive-behavioral techniques, mindfulness-based interventions, or acceptance and commitment therapy (ACT) in addressing the broader spectrum of psychological distress commonly seen in tinnitus patients (Husain et al., 2019; McKenna et al., 2020; Reeves et al., 2021).

By adopting a multidisciplinary approach that combines audiological, psychological, and therapeutic interventions, healthcare providers can better support individuals in managing both the auditory and emotional aspects of tinnitus. In this regard, counseling, support groups, and interventions aimed at enhancing resilience and coping strategies in the face of chronic tinnitus—related distress. During patient counseling, it is essential to recognize the patient’s frequent desire to eliminate the tinnitus immediately and it is essential to communicate that this expectation is sometimes unreachable and may exacerbate the perception of tinnitus intrusiveness. The improvement in tolerance of tinnitus seems to be related to the reduction of constant worry about tinnitus. According to the empowerment model (Dauman and Dauman, 2021), a complete dedication to meaningful goals could help patients mitigate tinnitus-related complaints, cultivating a sense of responsibility in the management of debilitating tinnitus. Hence, the combination of psychotherapy with existing tinnitus interventions may enhance outpatients’ experience, consistent with interdisciplinary efforts that address both auditory and psychological aspects in healthcare.

Recent studies have shown that tinnitus severity and tinnitus perception are correlated with lower performance on tasks involving the deployment of cognitive functions (Neff et al., 2021). To this end, the MMSE was used to estimate global cognitive functioning, whereas the FAB was used as a screening test to assess executive function among patients with tinnitus. Just as cognitive and perceptual loads are likely to have a significant influence on both tinnitus perception and emotional well-being, cognitive resource overload in chronic tinnitus leads to failures in executive control tasks in accordance with the so-called “load theory” (Khan and Husain, 2020). On a phenomenological point of view, therefore, tinnitus-related discomfort is often expressed together with psychosomatic phenomena, especially somatization symptoms that may or may not occur in the context of identifiable medical factors such as dizziness, sweating, blurred vision, headache, periods of weakness, pain, nausea, or shortness of breath (Sahin et al., 2016). Thus, while the tinnitus sound might come from audiological or somatosensory factors, chronicity seems to develop along a cognitive-emotional trajectory (Wallhäusser-Franke et al., 2017) that likely involves complex interactions between psychological and somatic vulnerabilities.

Significant differences were found in MMSE scores between the tinnitus and control groups, while there was no association between equivalent MMSE scores and tinnitus-related severity levels. Our results showed that tinnitus can affect global cognitive functioning, with greater difficulties in carrying out simple tasks and worse performance on cognitive tasks, as suggested in a previous study (Clarke et al., 2020). Recent evidence indicates that sensory and motor changes may precede the cognitive symptoms of dementia over several years and may increase the risk of developing AD (Jafari et al., 2019). In particular, there is a strong link between annoying tinnitus and cognitive impairment in adults ranging from young adults to the elderly (Tegg-Quinn et al., 2016; Gudwani et al., 2017). This association stems from the fact that tinnitus is not only an aberrant auditory sensory perception but also related to a variety of non-auditory symptoms that lead to frustration and difficulty concentrating (Trevis et al., 2016). Among the participants in our tinnitus group, 80.7% presented with HL, which has been linked to cognitive decline (Sardone et al., 2021). Therefore, it is difficult to measure the selective effect of tinnitus compared with HL on cognitive decline.

Recent research includes hearing loss as one among many risk factors for dementia (Livingston et al., 2020), and age-related decline might be attributed to auditory impairment, even if this relationship requires further investigations to be elucidated (Powell et al., 2022). Age-related hearing loss (ARHL) ranks as the third most prevalent chronic disability among elderly individuals. Research indicates that ARHL is linked to a higher likelihood of cognitive decline and the development of dementia (Jafari et al., 2019).

Even if hearing loss might be associated with cognitive impairment in older people, this association has not claimed to be direct. Despite numerous research efforts, elucidation of the underlying causal relationships between auditory and cognitive decline has not yet reached a consensus (Uchida et al., 2019) but may also depend on other factors associated with hearing loss such as social isolation, poorer lifestyle (Swain, 2021) and cognitive reserve (Livingston et al., 2020). Indeed, it should be noted that individuals with hearing impairment may experience further conditions, including the potential impact of social factors like loneliness (Hackett et al., 2023), and that some dementing conditions can lead to changes in auditory processing (Ruan et al., 2023). As well, tinnitus, another persistent auditory condition, becomes more prevalent as individuals grow older (Jafari et al., 2019), but there was no direct association that still needs to be further investigated.

Among several cognitive domains, executive functions, involved in activities requiring the coordination of multiple tasks simultaneously, seem to be most sensitive to normal aging or chronic physical conditions (Bherer, 2015). Excessive cognitive load devoted to auditory perceptual processing in everyday life causes relevant structural changes in the brain and neurodegeneration at the expense of other cognitive processes (Uchida et al., 2019). Concerning executive functions, literature suggests that tinnitus is associated with worse performance in tasks subtending executive functions. Working memory skills, inhibitory control, cognitive flexibility, and processing speed may also be impaired (Clarke et al., 2020; Waechter et al., 2021).

In this study, no statistically significant differences in FAB scores were found between patients with tinnitus and the control participants. Therefore, the presence of tinnitus does not have a specific impact on a person’s performance compared with healthy people. However, the FAB scores correlated with the severe and catastrophic THI scores.

Most previous studies have examined differences in cognitive performance between individuals with and without tinnitus, while only a few studies have reported exploratory correlations with tinnitus measures. In addition, the results of studies that have investigated the correlation between frontal function and tinnitus severity are generally indicative of reduced cognitive performance in severe tinnitus, particularly in attention, processing speed, and executive functions (Cardon et al., 2019; Rosemann and Rauschecker, 2022); however, there are also reports of absent or conflicting results (Trevis et al., 2016; Waechter et al., 2021).

It is imperative to emphasize that our study meticulously incorporated an extensive array of psychological and cognitive measures, encompassing assessments of SCL-90 scores, BDI scores, FAB evaluations, and MMSE values, with the explicit aim of elucidating their potential impact on the Tinnitus Handicap Inventory (THI) levels within the tinnitus patient cohort. However, our findings consistently manifested an absence of statistical significance within these associations.

These null findings engender several noteworthy implications. Firstly, they allude to the intricate and multifaceted nature of the relationships between tinnitus-related distress, as quantified by the THI scores, and the aforementioned psychological and cognitive variables. Secondly, these results prompt consideration of the potential involvement of unmeasured or more nuanced factors that may underlie the determination of tinnitus-related distress severity.

Regarding the exclusion of SCL-subscales in the regression model used, it should be noted that our choice lied in the necessity to prevent subdomains from SCL-90-R (such as anxiety, depression and somatization) from overlapping those from more specified and targeted tools like STAI-Y, BDI and DCPR.

Moreover, the absence of statistically significant associations among the specific variables investigated here accentuates the necessity for further exploration and deliberation regarding additional factors that may contribute to the emergence and severity of tinnitus-related distress. This may depend for instance on several factors, including the relatively small sample size, the influence of unaccounted variables that were not expressed in the available clinical data, and the omission of other outcome measures in predicting the Tinnitus Handicap Inventory (THI). It is essential to note that while THI is the singular index considered for assessing the perceived intensity of tinnitus, it serves as an indicator of the genuine impact of the symptom on daily quality of life (Monzani et al., 2008).

While our investigation did reveal a heightened prevalence of psychosomatic symptoms within the tinnitus patient group compared to the healthy counterparts, it is noteworthy that tinnitus patients appear more inclined to manifest a broader spectrum of psychosomatic syndromes. This observation is particularly evident when contrasted with healthy individuals who either report no somatic symptoms or, at most, a solitary pathological condition. However, concerning to specific dimensions of the DCPR, namely irritability, somatization, and AIB, no statistically significant correlations emerged concerning tinnitus severity as measured by the THI.

This seemingly paradoxical discovery underscores a pivotal point: the intensity of tinnitus perception is not primarily determined by any singular psychosomatic dimension, nor does it exert a unilateral influence in return. Rather, the perceived intensity of tinnitus is intricately intertwined with a greater multitude of both psychological and somatic symptoms. This observation reiterates the intricate nature of tinnitus and the multifaceted interplay of variables within this clinical context (Mohan et al., 2022).

Once more, it underscores the imperative for further investigation in this field, aiming to discern the nuanced influence of specific factors. Despite the wealth of scientific evidence addressing the psychological manifestations in tinnitus patients, our findings underscore the need for continued research endeavors capable of disentangling the intricate web of variables at play in this complex clinical landscape.

Despite being preliminary, this study included, besides the evaluation of psychological symptoms, a screening of psychosomatic conditions and cognitive functioning in tinnitus patients, using structured procedures suitable for the Italian population (as for DCPR), and incorporating a matched control group. This approach might provide a more structured framework to understand the relationship between psychological distress, cognitive status, multicomponent psychosomatic symptomatology, and tinnitus severity, offering a starting point for integrating patient assessment and treatment protocols.

The identification of cognitive, psychological, and psychosomatic factors and the focus on potential predictors involved in tinnitus experience warrant further investigation that may elucidate the underlying mechanisms and explore potential interventions targeting them. Considering the involvement of psychosomatic conditions in tinnitus, interventions that focus on psychosomatic symptom management and psychological well-being could be explored to improve the overall quality of life for individuals with tinnitus, especially those reporting high severity. Additionally, longitudinal studies tracking the progression of tinnitus and the dynamics of psychosomatic conditions and cognitive functioning over time may provide valuable insights into the development of timely interventions for tinnitus patients.

The main limitation of the present study is the absence of audiometric data for the control group. We propose that this study represents the first step in terms of understanding the impact of hearing loss on the psychological and cognitive spheres, as many studies on tinnitus do not report audiometric data (Mohamad et al., 2016). Another limitation might be the cross-sectional study design; these types of studies are generally inexpensive and easy to conduct, assess exposure and outcome simultaneously, and therefore, a true cause-and-effect relationship cannot be established as in longitudinal studies. Moreover, regarding the sample size, a power analysis was performed using G Power 3.1.9.7, to determine the effect sizes that our sample size can adequately detect with a power of 80% and an alpha-error probability of less than 5%. The estimated sample size for this study is 64 participants. Indeed, it was influenced by the outcomes of comprehensive assessments. The real number of participants (62) approximates this size but does not exactly match it. Therefore, the results appear preliminary and warrant validation on larger samples.

In conclusion, our results demonstrate the importance of focusing on the characteristics of early emotional, psychosomatic, and cognitive disturbances in tinnitus patients as potential predictors of significantly impaired functioning in these patients. We suggest the adoption of reliable tools such as the THI and cognitive and psycho-diagnostic screening tests during the audiological interview to refer patients for specific neuropsychological evaluation, resulting in a more accurate diagnosis with a multidisciplinary approach and more effective therapy.

## Data availability statement

The raw data supporting the conclusions of this article will be made available by the authors, without undue reservation.

## Ethics statement

The studies involving humans were approved by Interregional Ethics Committee of the “Azienda Policlinico” of Bari. The studies were conducted in accordance with the local legislation and institutional requirements. The participants provided their written informed consent to participate in this study. Written informed consent was obtained from the individual(s) for the publication of any potentially identifiable images or data included in this article.

## Author contributions

DG: Data curation, Investigation, Methodology, Writing – original draft. IP: Formal analysis, Investigation, Methodology, Writing – review & editing. DL: Conceptualization, Formal analysis, Methodology, Writing – original draft. CA: Conceptualization, Investigation, Methodology, Writing – original draft. MD: Conceptualization, Supervision, Validation, Writing – review & editing. AT: Conceptualization, Supervision, Validation, Writing – review & editing. DD’E: Data curation, Investigation, Writing – review & editing. PF: Conceptualization, Methodology, Supervision, Writing – review & editing. LA: Conceptualization, Formal analysis, Methodology, Supervision, Writing – review & editing. AP: Data curation, Investigation, Writing – review & editing. GC: Data curation, Writing – review & editing. VP: Data curation, Writing – review & editing. PT: Conceptualization, Investigation, Methodology, Project administration, Supervision, Writing – review & editing. NQ: Conceptualization, Funding acquisition, Resources, Writing – review & editing.
