# Supplementary material for: Cognitive functioning and psychosomatic syndromes in a subjective tinnitus sample
Source: Front Psychol. 2023 Dec 20;14:1256291. doi: 10.3389/fpsyg.2023.1256291 (PMC10773809; doi:10.3389/fpsyg.2023.1256291)
Supplement: Supplementary file 1 [file Table_1.pdf]

Supplementary Material

1 Supplementary Data

Table 1. Correlation coefficients between predictors of the regression model

	BDI	DCPR_0_1	IRRITABILITY	SOMATIZATION	AIB	MMSE	FAB	TINNITUS DURATION	STAI_Y1	STAI_Y2	THI
**STAI_Y1**											
Pearson correlation	.513	.223	.081	.141	.080	-.350	.127	.005			.322
Sig. (2-tailed) with Benjamini-Hocberg correction	.002	.086	.536	.283	.543	.008	.335	.972			.026
N	60	60	60	60	60	60	60	60			59
**STAI_Y2**											
Pearson correlation	.686	.168	.084	.214	.064	-.361	-.103	.036			.500
Sig. (2-tailed) with Benjamini-Hocberg correction	.008	.402	.696	.269	.718	.02	.692	.783			.000
N	60	60	60	60	60	60	60	60			59
**BDI**											
Pearson correlation		.334	.207	.305	.105	-.261	-.132	-.081	.513	.686	.467
Sig. (2-tailed) with Benjamini-Hocberg correction		.027	.163	.038	.475	.075	.401	.537	.000	.000	.000
N		61	61	61	61	61	61	61	60	60	60
**DPCR_0_1**											
Pearson correlation	.334		.348	.348	.609	-.023	.118	.008	.223	.168	.346
Sig. (2-tailed) with Benjamini-Hocberg correction	.025		.018	.018	.000	.949	.464	.949	.154	.301	.02

N	61		62	62	62	62	62	62	60	60	61
**IRRITABILITY**											
Pearson correlation	.207	.348		.073	.128	-.137	-.149	.157	.081	.084	.221
Sig. (2-tailed) with Benjamini-Hocberg correction	.327	.054		.571	.320	.411	.411	.411	.571	.327	.145
N	61	62		62	62	62	62	62	60	60	61
**SOMATIZATION**											
Pearson correlation	.305	.348	.073		.055	.032	.107	-.085	.141	.214	
Sig. (2-tailed) with Benjamini-Hocberg correction	.076	.054	.734		.757	.802	.732	.734	.636	.303	
N	61	62	62		62	62	62	62	60	60	
**AIB**											
Pearson correlation	.105	.609	.128	.055		.005	.051	.104	.080	.064	-.096
Sig. (2-tailed) with Benjamini-Hocberg correction	.696	.000	.696	.696		.696	.696	.696	.696	.696	.512
N	61	62	62	62		62	62	62	60	60	61
**MMSE**											
Pearson correlation	-.261	-.023	-.137	.032	.005		.261	-.163	-.350	-.361	-.321
Sig. (2-tailed) with Benjamini-Hocberg correction	.090	.858	.287	.858	.858		.090	.370	.027	.027	.026
N	61	62	62	62	62		62	62	60	60	61
**FAB**											
Pearson correlation	-.132	.118	-.149	.107	.051	.261		.118	.127	-.103	-.152
Sig. (2-tailed) with Benjamini-	.487	.487	.487	.487	.691	.487		.487	.487	.487	.345

Hocberg correction											
N	61	62	62	62	62	62		62	60	60	61
**TINNITUS DURATION**											
Pearson correlation	-.081	.008	.157	-.085	.104	-.163	.118		.005	.036	.081
Sig. (2-tailed) with Benjamini-Hocberg correction	.805	.972	.805	.805	.805	.805	.805		.972	.972	.537
N	61	62	62	62	62	62	62		60	60	61

Acronyms: BDI=Beck Depression Inventory; DCPR= Diagnostic Criteria for Psychosomatic Research; DPCR_0=Absence of Diagnostic Criteria for Psychosomatic Research; DPCR_1= At least one disorder of Diagnostic Criteria for Psychosomatic Research. IRRITABILITY= Irritability Cluster of Diagnostic Criteria for Psychosomatic Research; SOMATIZATION= Somatization Cluster of Diagnostic Criteria for Psychosomatic Research; AIB= Abnormal Illness Behaviour; MMSE= Mini-Mental State Examination; FAB= Frontal Assessment Battery; STAI Y1= State-Trait Anxiety Inventory Y1; STAI Y2= State-Trait Anxiety Inventory Y2; THI= Tinnitus Handicap Inventory.
